# Epigallocatechin-3-Gallate Delivery in Lipid-Based Nanoparticles: Potentiality and Perspectives for Future Applications in Cancer Chemoprevention and Therapy

**DOI:** 10.3389/fphar.2022.809706

**Published:** 2022-04-14

**Authors:** Fulvia Farabegoli, Marina Pinheiro

**Affiliations:** ^1^ Department of Pharmacy and Biotechnology (FABIT), University of Bologna, Bologna, Italy; ^2^ LAQV, Rede de Química e Tecnologia (REQUIMTE), University of Porto, Porto, Portugal; ^3^ Life and Health Sciences Research Institute (ICVS), School of Medicine, University of Minho, Braga, Portugal

**Keywords:** cancer, catechins, medicinal chemistry, nanomedicine, nutraceutics, EGCG

## Abstract

Chemoprevention is a strategy aimed to not only reduce the risk but also delay the development or recurrence of cancer. An ideal chemopreventive agent is not dangerous and ought not to result in side effects or damage to human health. In this context, epigallocatechin-3-gallate (EGCG) is considered a suitable chemopreventive agent, but its clinical use is limited by many factors, namely, the difference in source, administration, individual metabolism, absorption, and distribution. Genetic and dietary differences greatly cause this variability, which has limited the rational use of EGCG in chemoprevention and, particularly, the definition of a safe and efficient concentration. In the present mini review, the main limitations to a complete understanding of the use of EGCG as a chemopreventive agent will be briefly illustrated. This review also indicates the introduction and trialing of lipid-based nanoparticles (NPs) as a proper strategy to deliver EGCG at a well-defined concentration for better investigation of the chemopreventive activity. Finally, some examples of cancers that might benefit from EGCG treatment in different stages of the disease are proposed.

## Introduction

EGCG, the most abundant catechin in green tea, is considered a suitable chemopreventive agent based on epidemiologic (Yuan et al., 2011; Yi et al., 2019; Zhao et al., 2021) and animal model studies (Khan and Mukhtar, 2007; Yang et al., 2011; Yiannakopoulou, 2014). *In vitro* studies on human cancer cells have also provided a large set of data that indicate the cytotoxicity of EGCG on cancer cells (Singh et al., 2011; Gan et al., 2018; Shirakami and Shimizu, 2018; Aggarwal et al., 2020). On the other hand, many contradictory results have also been published, and doubts on its potential use in humans exist (Filippini et al., 2020; Kim et al., 2020). The complexity of unraveling the activity of EGCG against cancer cells is due to the very high number of variables, which are difficult to interpret correctly (Mereles and Hunstein, 2011). A first issue concerns the poor physicochemical stability and low bioavailability of EGCG (Lambert and Yang, 2003; Chow and Hakim, 2011; Sang et al., 2011). Oral consumption of EGCG bioavailability after oral intake can be reduced or increased by assuming various food and beverages (Kale et al., 2010; Peters et al., 2010; Naumovsky et al., 2015). Thus, EGCG, which is only partly degraded in the stomach at low pH, reaches the intestine, where the pH is neutral-alkaline, and is further degraded (Neilson et al., 2007). The quantity of EGCG that crosses the enterocytes is low: EGCG enters the enterocytes mainly by passive diffusion since no specific receptors carrying EGCG exist on the surface of enterocytes. Active outflow by multidrug resistance-associated protein 2 (MRP2) may occur, further lowering EGCG absorption (Vaidyanathan and Walle, 2001; Hong et al., 2003; Scholl et al., 2018). Once into the enterocytes, the EGCG is actively metabolized by phase II enzymes, conjugated with glucuronic acid and sulfate, or by methylation or methylated by catechol-Omethyltransferase (COMT). Glucuronidation and sulfation mainly occur in the intestine, whereas glucuronidation, sulfation, and methylation occur later in the liver (Singh et al., 2011). Some conjugates are further methylated. Genetic heterogeneity due to a polymorphism involving COMT results in a low-activity variant, which possesses a 40–75% less catalytic capacity than the enzyme coded with wild-type alleles and can introduce a high variability in EGCG activity (Wu et al., 2003; Miller et al., 2012; Lai et al., 2019). A large part of orally taken EGCG is effluxed from the enterocytes into the intestinal lumen or from the liver to the bile and excreted in the feces, and hence lost. Gut microbiota plays a critical role in the metabolism of EGCG. Microbiota can deconjugate and degrade EGCG (Zhang et al., 2013; Liu et al., 2020). In contrast, it has been found that metabolites of green tea, including EGCG, produced by gut microbes, have significant health benefits: small molecules derived from breakdown can show antioxidant and anti-inflammatory capacity, correct dysbiosis, and decrease harmful metabolites (Zhang et al., 2019; Xu et al., 2020). Then, ultimately, microbiota may concur to improve the health effects of EGCG. In addition to cancer cell cytotoxicity, indirect advantages of EGCG on cancer onset and development are also related to the metabolic (antidiabetic and contrasting obesity effects) (Hursel et al., 2009; Hara-Terawaki et al., 2017; Quezada-Fernández et al., 2019), antioxidative, and anti-inflammatory actions of EGCG (Chen et al., 2018), all these conditions being clearly associated with cancer development (Thielecke and Boschmann, 2009; Yang et al., 2016; Oz, 2017; Potenza et al., 2020).

Then, why should EGCG be proposed as a chemopreventive agent? The relative potency of all these variables to stability and bioavailability makes defining the health effects of green tea catechins and EGCG unpredictable. Several observational and interventional studies have been reviewed extensively over the last years (Clement, 2009; Fujiki et al., 2018; Almatroodi et al., 2020). The findings are conflicting, but a consistent number of studies on several human malignant neoplasia support the potential use of EGCG as a chemopreventive agent despite this variability: any improvement in EGCG stability and bioavailability is, therefore, supposed to improve the efficacy. When we turn to animal models, there is a general agreement that a clear chemopreventive effect independent of the experimental model used (chemical carcinogenesis, xenograft tumors, knock-out animals spontaneously developing cancer, etc.) occurs. A large majority of studies clearly demonstrated that EGCG, usually used as a beverage, delayed cancer onset and the metastatic process and reduced the size and number of neoplastic foci (Ju et al., 2007; Fujiki et al., 2018; Gan et al., 2018). In the context of animal models, the number of variables under evaluation and concurring to the final target decreases: animals are genetically related, frequently inbred, exposed to the same environmental conditions that are controlled from the beginning to the end of the experimental treatments, and consume the same food, which is very simple and not varied. In these conditions, EGCG shows chemopreventive efficacy (Ju et al., 2007; Yang et al., 2011).

Another conflicting point concerns “*in vitro*” experiments on cell lines. In this case, the EGCG concentrations used to obtain significant results, including cytotoxicity and downregulation of molecular pathways involved in cancer onset and development, are far from those measured *in vivo*, in blood and tissues, after EGCG oral administration (Gan et al., 2018; Almatroodi et al., 2020). This discrepancy seems to be related to the fact that EGCG dissolved in culture medium is barely taken up by cells and is unstable at pH greater than 5 (Hong et al., 2002). Furthermore, oxidation processes may occur, and they are considered misleading with respect to the interpretation of the final cytotoxic effects (Lambert and Elias, 2010; Wei et al., 2016). On the other hand, currently a large number of studies are underway on human cancer cell lines: extensive meta-analysis in the last years reported coherent results concerning the molecular targets (Singh et al., 2011; Gan et al., 2018; Shirakami and Shimizu, 2018; Aggarwal et al., 2020; Farooqi et al., 2020) and stressed another crucial point: EGCG is safe for normal cells since very high concentrations can only induce cell death (Weisburg et al., 2004; Papi et al., 2013; Tyagi et al., 2015; Luo et al., 2018; Ni et al., 2018; Xie et al., 2018). So, EGCG is preferentially taken up by cancer cells rather than by normal cells, fulfilling one of the basic characteristics of a molecule suitable for chemoprevention. Can the potentiality of EGCG as a chemopreventive agent be improved in view of future applications?

## Application of Nanotechnology in Epigallocatechin-3-Gallate Delivery Systems to Cancer Cells

The use of nanotechnology, namely, the development of drug delivery systems, especially biocompatible nanoparticles (NPs), constitutes a promising approach to increase the bioavailability and stability of this natural compound (Granja et al., 2017; Cai et al., 2018; Granja et al., 2019). Different types of NPs, including lipid-based NPs, polymeric NPs, and gold NPs, among others, can be used, and for more comprehensive information, the reader can consult the following studies: Min and Kwon (2014), Granja et al. (2017), and Li et al. (2020). NPs are extremely versatile and can be easily modified at their surface to selectively target cancer cells and, therefore, selectively release ECGC, especially inside tumor cells (Li et al., 2020). The main ligands that are used include antibodies that recognize tumor cells and folic acid and analogs because of the higher expression of folic acid receptors in cancer cell lines than in healthy cells (Granja et al., 2017). Among the different types of NPs, lipid-based NPs emerge as the most promising drug delivery systems because their composition is based on lipids, which also exist in the human body, making the NPs biocompatible and biodegradable (Nature Reviews Materials, 2021). One important advantage of their use is also their production simplicity, which is based on nanoemulsion oil/water production, and their high physicochemical stability, which is demanding for their scale-up (Frias et al., 2016).

### Lipid-Based Nanoparticles

Lipid-based NPs constitute a broad and diverse group that includes liposomes and lipid NPs (Garcia-Pinel et al., 2019). Liposomes were discovered in 1965; are vesicular structures, constituted by phospholipid bilayers, enclosing an aqueous medium; and have been attracting interest as nanocarriers for many years (Bangham et al., 1965a; Bangham et al., 1965b). Thus, liposomes are already used in the clinical industry in several marketed formulations, including Doxil^®^, Ambisome^®^, and DepoDur™, among others (Bulbake et al., 2017). Solid lipid nanocarriers (SLNs) were developed in the 1990s, and next-generation nanostructured lipid carriers (NLCs) found almost 10 years later to improve the stability and capacity loading of SNLs (Naseri et al., 2015). The lipid NPs approved for clinical use were recently used in COVID-19 mRNA vaccines (Nature Reviews Materials, 2021). This mini review highlights the main contributions of EGCG lipid-based NPs developed in recent years and applied in cancer therapy and their enormous advantages and potential in clinical use. The main characteristics of lipid-based NP EGCG delivery systems in cancer therapy are summarized in Table 1. In 2005, Fang et al. developed liposomes loaded with EGCG of 157 nm diameter and with an encapsulation efficiency (EE) of 57%. The authors demonstrated that the formulation allowed the accumulation of EGCG in the tissues of Balb/c-nu, a mouse model of basal cell carcinoma. One year later, the authors developed liposomes of size 100 nm and with an EE of 100%. The authors demonstrated that in comparison with unloaded EGCG, the liposomal formulations demonstrated a 20-fold higher EGCG deposition (Fang et al., 2005; Fang et al., 2006). In 2013, Cohen de Pace et al. developed nanoliposomes loaded with EGCG of diameter 56 nm and with an EE of 90% and demonstrated that this formulation significantly enhanced EGCG stability, improved its sustained release, and increased the intracellular EGCG content in MCF7 cells, inducing apoptosis of MCF7 cells and inhibiting MCF7 cell proliferation compared to unloaded EGCG. In addition, the authors demonstrated that the developed formulation retained its antiproliferative and proapoptotic effectiveness at 10 µM or lower, at which unloaded EGCG does not have any beneficial effects (Cohen de Pace et al., 2013). In 2015, Ramadass et al. developed liposomes with EGCG of diameter 127 nm and with an EE of 59%. They demonstrated the synergistic outcome of a combination of EGCG with an anticancer drug paclitaxel (PTX) in an MDA-MB-231 breast cancer cell line and concluded on the suitability of PTX/EGCG co-loaded liposomes for the treatment of invasive breast cancer (Ramadass et al., 2015). In 2016, Radhakrishnan et al. developed EGCG solid lipid NPs of diameter 157 nm and with an EE of 67%. This formulation was tested in MDA-MB-231 and DU-135 cell lines, and an increase in cytotoxicity of 8.1- and 3.8-fold, respectively, was observed. In the same year, Frias et al. developed SLNs and NLCs loaded with EGCG of diameter 300–400 nm that demonstrated a higher EE of 80% and 90%, respectively, and a high stability during long-term storage (Radhakrishnan et al., 2016). Later, in 2019, Granja et al. developed NLCs of 300 nm diameter and with an EE of 90% and functionalized the NPs with folic acid to increase their transport across the intestinal barrier to reach the cancer cells (Granja et al., 2019). Recently, Radhakrishnan et al. developed solid lipid NPs loaded with EGCG and functionalized the NPs with a gastrin-releasing peptide receptor (GRPR)-specific peptide as GRPRs are overexpressed in breast cancer. The “*in vivo*” studies performed on C57/BL6 mice showed greater survivability and reduction in tumor volume in mice treated with functionalized solid lipid NPs than in mice treated with a nonfunctionalized formulation or with unloaded EGCG (Radhakrishnan et al., 2019). Table 1 summarizes examples of successful nanoformulations with EGCG. An update on this topic can be found in Yang et al. (2020), Kazi et al. (2020), and Rashidinejad et al. (2021).

**TABLE 1:** Lipid-based NPs, including liposomes and lipid NPs, as EGCG delivery systems.

Nanodelivery system	Particle size (nm)	EE (%)	Type of cancer	Primary outcome	References
Liposomes	157	57	Skin	Accumulation of EGCG in the tissues in a mouse model of basal cell carcinoma	Fang et al. (2005)
Liposomes	100	100	Skin	Liposomes allowed a higher EGCG accumulation within tumor cells	Fang et al. (2006)
Liposomes	56	90	Breast	Antiproliferative and proapoptotic effect in MCF7 breast cancer cells	Cohen de Pace et al. (2013)
Liposomes	127	59	Breast	Synergistic outcome of EGCG combination with an anticancer drug PTX in an MDA-MB-231 breast cancer cell line	Ramadass et al. (2015)
Solid lipid NPs	157	67	Breast and prostate	The NPs increase the cytotoxicity of MDA-MB-231 and DU-135 cell lines by 8.1- and 3.8-fold, respectively, in comparison with unloaded EGCG	Radhakrishnan et al. (2016)
Nanostructured lipid NPs	300	90	Not specified	Folic acid to make it easier for NLC-based nanoparticles to transport EGCG across the intestinal barrier to reach the cancer cells	Granja et al. (2019)
Solid lipid NPs	163	67	Breast	C57/BL6 mice showed greater survivability and reduction in tumor volume in mice treated with functionalized lipid NPs as compared to those treated with a nonfunctionalized formulation or with unloaded EGCG	Radhakrishna et al. (2019)

EGCG, epigallocatechin gallate; NPs, nanoparticles; NLCs, nanostructured lipid carriers; PTX, paclitaxel; SLNs, solid lipid nanoparticles; EE, encapsulation efficiency.

## Potential Applications and Key Issues for Further Research

Neoplastic disease is extremely heterogeneous, and even in the same tissue and organ, the multiplicity of cancer types having different degrees of malignancy and evolution is very high. Chemoprevention is classified as primary, secondary, or tertiary. *Primary chemoprevention* aims at preventing the development of premalignant lesions (often assessed by appropriate markers) and subsequent cancer in high-risk cohorts. *Secondary chemoprevention* prevents the evolution of premalignant markers/lesions into cancer. Finally, *tertiary chemoprevention* prevents the recurrence of cancer (Landis-Piwowar and Iyer, 2014).

### Premalignant Lesions in Sporadic Cancer

Sporadic cancer represents the most common human cancer. In some cases, premalignant lesions precede the development of frankly malignant neoplasms with invasive growth. Here, we discuss some aspects of a few neoplasms largely found in the human population, for example, prostatic intraepithelial neoplasia (PIN) and high-grade PIN in prostatic cancer, atypical hyperplasia and lobular/ductal carcinoma *in situ* (LCIS, DCIS) of the breast, and colon premalignant lesions, which can be identified by screening or are detected occasionally, and some open questions that might be addressed by a chemopreventive intervention based on EGCG. In some cases, surgery is the first option, but evolution of a premalignant lesion is difficult to predict and the risk of overtreatment must be evaluated too (Curtius et al., 2017). For a critically reviewed list of premalignant diseases potentially subject to chemopreventive interventions, the reader can consult Maresso et al. (2015). Chemoprevention might be an option to be investigated in all those cases where surgery is not considered necessary or a second-line intervention after surgery to target potentially evolving situations. A successful example of a green tea catechin (GTC) extract, orally administered in patients with high-grade PIN, was reported by Naponelli and coworkers (Naponelli et al., 2017). After 1 year of treatment, only one tumor was diagnosed among 30 GTC-treated men, while nine cancers were found among 30 placebo-treated men. Despite the limitations of stability and bioavailability of GTCs, many successful trials on the use of green tea or EGCG in prostate cancer prevention have been reported and critically analyzed (Guo et al., 2017; Jacob et al., 2017; Perletti et al., 2019).

In breast atypical hyperplasia and LCIS or DCIS, tamoxifen and raloxifene have been demonstrated to reduce breast cancer risk, but they must be used in postmenopausal women, reducing the number of patients who might obtain significant risk reduction benefits without incurring serious harm (Moen and Keating, 2008; Sauter, 2018). Aromatase inhibitors, which increase osteoporosis risk, are also preferentially used in postmenopausal women, but estrogen-insensitive breast neoplasms do not respond to this kind of treatment (Trivedi et al., 2017; Thorat and Balasubramanian, 2020). Therefore, premenopausal women and women with estrogen-negative neoplasms cannot benefit from this plan of chemoprevention. Patients with premalignant lesions, especially those in premenopause, might be eligible for a chemoprevention trial based on EGCG or green tea extract. In early breast carcinoma, EGCG administration 4 weeks before the surgery resulted in a higher concentration of EGCG in the tumor than in the adjacent normal tissue, which correlated with a lower Ki-67 index with respect to untreated patients (Lazzeroni et al., 2017). Mammographic density was found to be reduced by GTE supplementation in women at high risk of breast cancer, similar to tamoxifen treatment (Samavat et al., 2017). On the basis of numerous studies indicating a protective effect of green tea catechins in breast cancer development (Yu et al., 2019), EGCG nanoformulations might have the potential to reduce the risk in patients who develop premalignant breast lesions, independent of the presence or absence of estrogen receptors and age.

Colorectal cancer (CRC) is one of the leading causes of cancer deaths in the world (Arnold et al., 2017). Although recommendations for aspirin-based chemoprevention strategies have recently been established, hazards of the long-term use of aspirin make identification of individuals for whom the protective benefits outweigh the harms important (Katona and Weiss, 2020). Some premalignant diseases develop in the context of inflammatory bowel diseases (Nadeem et al., 2020) and require surgery to alleviate symptoms, independent of possible future cancer, such as colectomy for patients suffering from severe colitis symptoms. EGCG might be suggested as a chemoprevention strategy, particularly in patients suffering from severe colitis, due to the anti-inflammatory property of EGCG (Sokolosky and Wargovich, 2012; Wang et al., 2020). The development of a suitable nanoformulation for proper delivery to the colic mucosa might be an achievable target for these patients and an opportunity to delay and reduce cancer risk.

Disease-free intervals between chemo- and radiotherapy treatments occur in many cancer patients. In these phases, patients often do not receive any support (apart from some nutrition suggestions) to achieve remission or to prevent relapse. Compounds such as EGCG that have anti-inflammatory, antioxidant, and chemopreventive properties might also offer an opportunity of treatment for all patients experiencing undesired consequences of therapies. A special mention concerns childhood neoplasms: treatments in young cancer patients may open the way to secondary neoplasms that have time to insurge. Especially for very young cancer survivors, the option of safe intervention is mandatory (Gebauer et al., 2019).

## Conclusion

This mini review summarized the main contributions of lipid-based NPs for EGCG delivery in cancer prevention and therapy and demonstrated that lipid-based NPs offer a great opportunity to increase the potential of EGCG as a chemopreventive and therapeutic agent. Adequate clinical trials to establish the safety and efficacy of nanoformulations are urgently needed to validate EGCG as a crucial component in experiencing a new innovative chemoprevention and treatment strategy for cancer.
